# Dual attachment of the buccinator muscle at the pterygoid hamulus: implications for pterygoid implant surgery

**DOI:** 10.1186/s40729-025-00663-1

**Published:** 2025-12-31

**Authors:** Tianyi Yang, Masahito Yamamoto, Motonobu Abe, Satoshi Ishizuka, Kei Kitamura, Kenta Abe, Gen Murakami, Shinichi Abe

**Affiliations:** 1 Department of Anatomy, Tokyo Dental College, Chiyoda-ku, Tokyo, Japan; 2 Department of Anatomy, Aichi Gakuin University School of Dentistry, 1-100 Kusumoto-Cho, Chikusa-ku, Nagoya, 464-8650 Aichi Japan; 3 Department of Histology and Developmental Biology, Tokyo Dental College, Tokyo, Japan; 4 Department of Phamacology, Tokyo Dental College, Tokyo, Japan; 5 Division of Internal Medicine, Iwamizawa Asuka Hospital, Iwamizawa, Hokkaido Japan

**Keywords:** Pterygoid implant, buccinator muscle, maxillary tuberosity, Bone volume fraction, Cadaveric anatomy

## Abstract

**Purpose:**

Pterygoid implants are a viable alternative to sinus-lifting procedures; however, their placement may risk damaging adjacent soft tissues. This study aimed to clarify the morphology of the buccinator muscle (Bu), particularly its attachment to the pterygoid hamulus, and to assess the risk of injury during implant surgery.

**Methods:**

Cadaveric dissection, histological analysis, and micro-computed tomography were performed. Bone morphometry was used to evaluate the maxillary tuberosity. Histological sections were analyzed to measure the distance between the Bu and the maxillary tuberosity, as well as to examine its attachment to the pterygoid hamulus.

**Results:**

Substantial individual variation was observed in the shape and bone density of the maxillary tuberosity, with some specimens exhibiting low bone volume fraction (BV/TV). The Bu was located immediately posterior to the tuberosity at the root of the pterygoid hamulus (mean: 0.61 mm), but more distant at the tip (mean: 2.37 mm). The muscle exhibited a dual mode of attachment: tendinous at the root and periosteal at the tip. Implant perforation near the root may therefore pose a higher risk of muscle injury.

**Conclusions:**

This study revealed a dual attachment of the buccinator muscle to the pterygoid hamulus and emphasized its close proximity to the maxillary tuberosity. Additionally, low BV/TV values in some specimens highlight the anatomical variability of this region. Understanding individual differences in bone structure and the precise location of soft tissue attachments is essential for safer and more predictable pterygoid implant placement.

## Background

The buccinator muscle (Bu) is a thin, quadrilateral muscle that is located between the maxilla and mandible, where it contributes to the lateral wall of the oral cavity. In anatomical textbooks, it is generally described as originating from the outer surfaces of the alveolar processes of the maxilla and mandible, as well as the pterygomandibular raphe, with its fibers merging into the orbicularis oris muscle at the angle of the mouth [1]. However, in his *Topographical Atlas of Human Anatomy* (1974) [2], Seihō Nishi reported that the Bu also attaches to the pterygoid process of the sphenoid bone. In contrast, the 32nd edition of *Gray’s Anatomy* (1958) [3] does not mention the pterygoid process as one of the origins of the Bu; instead, it describes how several Bu fibers arise from a fine tendinous band that bridges the interval between the maxilla and the pterygoid hamulus. Interestingly, this attachment is not present in contemporary anatomical textbooks [1], raising the question of whether it has been overlooked or omitted. If the Bu does indeed extend to the pterygoid process, this may have significant implications for our understanding of the biomechanical function, embryological origin, and clinical relevance of the Bu.

Recently, Iwanaga et al. [4]. challenged the traditional description of the Bu’s origin. Their study revealed that the Bu can be divided into three distinct parts, i.e., the maxillary tuberosity, the conjoint tendon of the temporalis, and the pterygoid hamulus; this account does not describe involvement of the pterygomandibular raphe. One major clinical implication of this revised anatomical understanding concerns the placement of pterygoid implants, which are used for prosthetic rehabilitation in cases of severe maxillary atrophy [5]. These implants are designed to anchor into the maxillary tuberosity and the pterygoid region of the sphenoid bone, thereby providing an alternative to bone grafting for edentulous patients [6–12]. However, the placement of the pterygoid implant is technically demanding due to the complex anatomy of this region, which includes critical neurovascular structures. In another study, Taniguchi et al. [13] investigated the anatomical risks associated with pterygoid implant placement, and highlighted the presence of vascular and neural structures passing through bony dehiscences in the greater palatine canal. If the observation of Nishi is correct, an additional, previously unrecognized risk may exist i.e., implant placement may potentially damage the Bu and its pterygoid attachment. Such damage may lead to altered muscle function, postoperative complications such as pain and fibrosis, or even impaired oral biomechanics. Furthermore, if correct awareness of this attachment should inform implant design and placement strategies to minimize soft tissue trauma.

Therefore, this study aims to reassess the anatomical attachments of the Bu, especially whether it connects to the pterygoid process. By re-examining historical and contemporary anatomical evidence, we seek to clarify whether this attachment should be reconsidered in anatomical education and clinical practice, particularly regarding maxillofacial surgery and implantology.

## Materials and methods

This study was performed in accordance with the provisions of the Declaration of Helsinki 1995 (as revised in Edinburgh 2013). Specifically, we first obtained approval from the Tokyo Dental College Ethics Committee (Approval No. 922-2), and subsequently used cadavers and skulls of Japanese origin that had been donated to Tokyo Dental College for research and education in human anatomy in all investigations. In all cases, the cause of death of cadavers used was ischemic heart or brain disease, and no macroscopic pathologies were evident in the head, thorax, or abdomen during dissection.

**Fig. 1:**
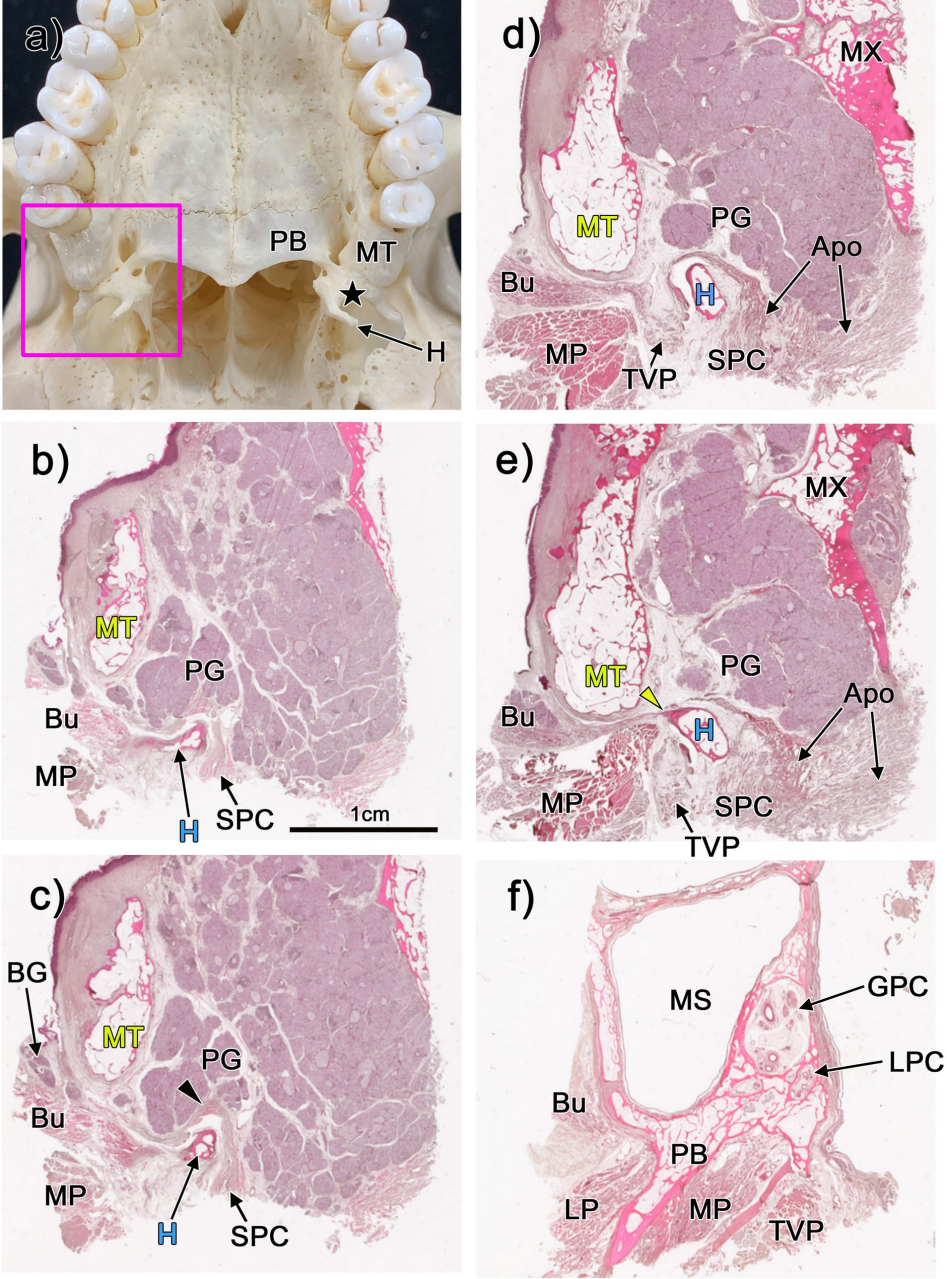
Topographical anatomy of the maxillary tuberosity and surrounding tissues. Panel (**a**) shows an inferior view of the maxillary tuberosity (MT) and surrounding tissues in a dry skull. The hamular notch, marked by a black star, lies between the MT and the pterygoid hamulus (H) (Panel a). Panels (**b**-**f**) show horizontal sections stained with HE, all at the same magnification (×2.5). Panel b (and f) shows the most inferior (and superior) planes present in the figure. Palatine glands (PGs) are found in the superficial layer of the hamular notch (shown in panels b, c). The buccinator muscle (Bu) and the medial pterygoid muscle (MP) are located posterior to the MT (panels b-e). The MP attaches to the pterygoid fossa (*n* = 4) (panel f) and the lateral pterygoid muscle (LP) attaches to the lateral pterygoid plate (*n* = 4) (panel f). The Bu contacts the lateral side of the MT (*n* = 4) (panel f)

After removing the brain, each cadaveric head was divided into left and right halves. We then dissected the hamular notch region between the maxillary tuberosity and the pterygoid hamulus in five of the donated cadavers (i.e., three males and two females, aged 72–91 years at death) (Fig. 3). To obtain histological samples, we removed the hamular notch region and its surrounding structures in four of the donated cadavers (i.e., two males and two females, aged 75–95 years at death; Figs. 1, 4 and 5). Following brain removal, the entire mandible was removed along with the masseter and medial pterygoid muscles. To do so, a horizontal cut was made above the maxillary sinus to obtain an almost cubic block of the hamular notch region. Before dissection, all cadavers had been fixed via arterial perfusion with a 10% v/v formalin solution and were then stored in a 50% v/v ethanol solution for more than three months. Tissues were decalcified via incubation in Plank–Rychlo solution (AlCl_3_·6 H₂O, 7.0 w/v%; HCl, 3.6; HCOOH, 4.6) at room temperature for 1–2 weeks. Following routine procedures for paraffin embedding [14–18], we prepared large horizontal and frontal sections at 200-µm intervals. Next, we obtained histological images using a Nikon Eclipse 80 microscope.

**Fig. 2:**
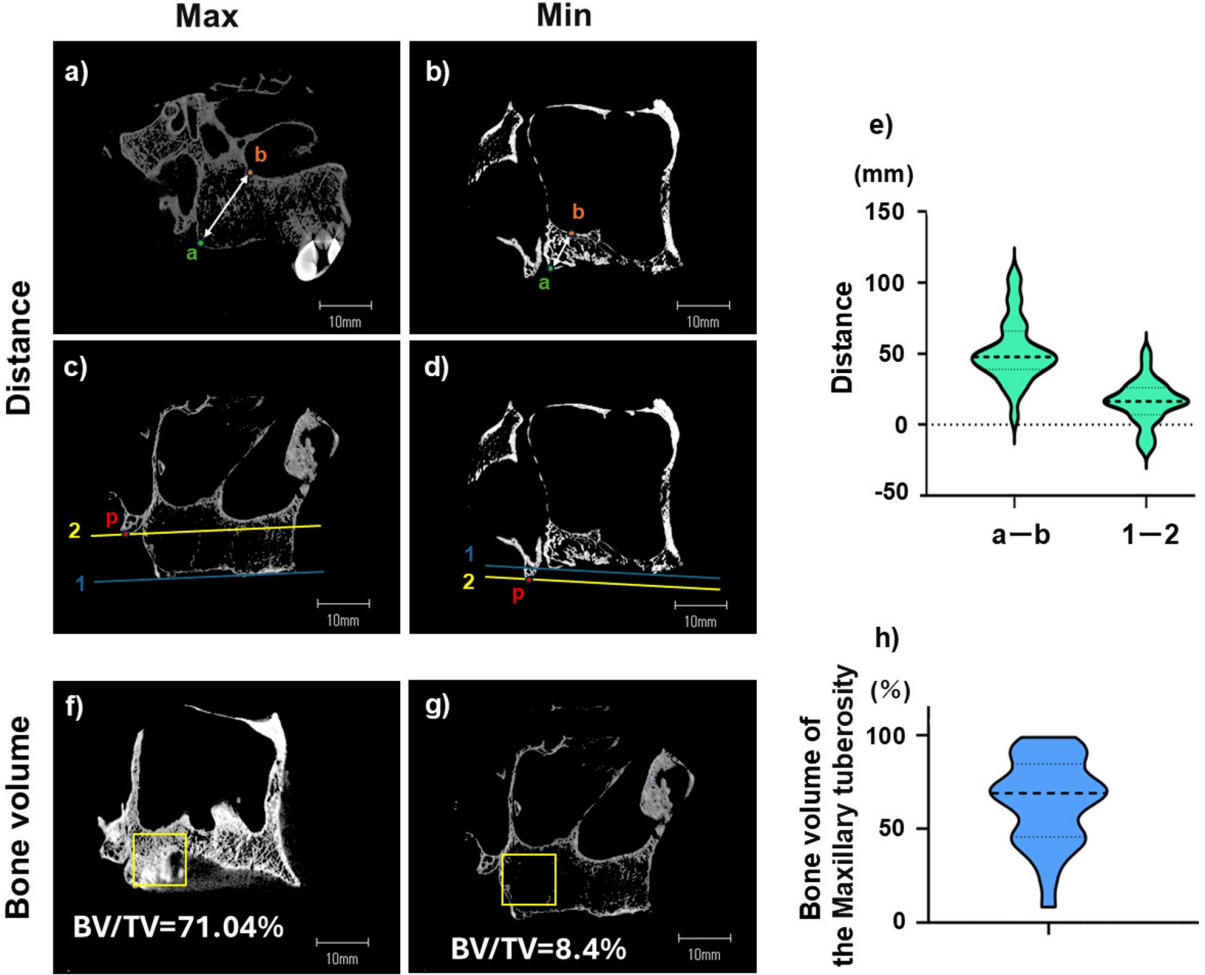
Measuring the bone morphology of the maxillary tuberosity. Panels (**a**, **b**) show the shortest distance between the posterior points of the maxillary tuberosity (MT) and the inferior margin of the maxillary sinus. Here panel (**a**) shows the maximum and panel (**b**) shows the minimum distance. Panels (**c**, **d**) show the MT-pterygoid hamulus (H) height difference. Here a greater difference increases implant perforation risk in the hamular notch. Panel (**c**): large difference, (**d**): MT superior to H (height reversal). Panel (**e**) illustrates the considerable individual variability in these parameters observed between the posterior points of the MT and the inferior margin of the maxillary sinus (n=31). Panel (**f**) shows the MT with a high BV/TV value, while Panel (**g**) shows the maxillary tuberosity (MT) at a low BV/TV value. The graph shown in Panel (**h**) demonstrates the considerable individual variability in BV/TV values within the MT (n = 31).

**Fig. 3:**
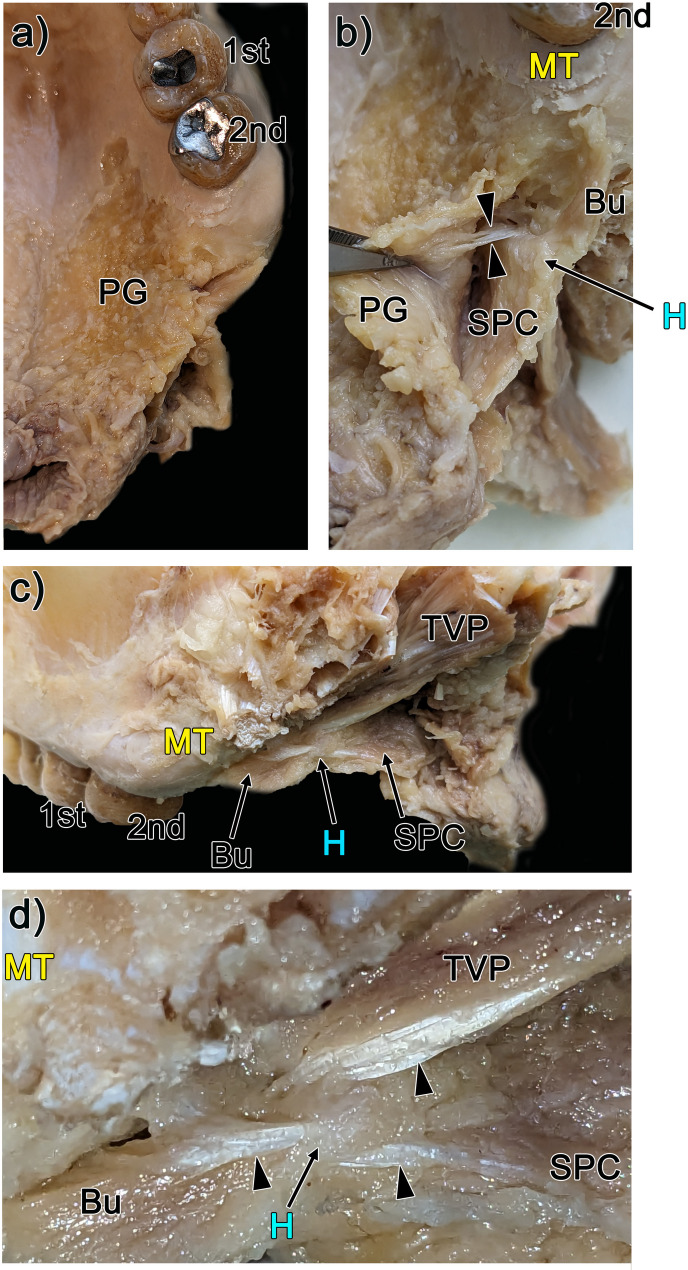
Gross anatomy of the hamular notch region between the maxillary tuberosity and the pterygoid hamulus. Panel (**a**): Palatine glands (PG) extend across the hard and soft palates. Panel (**b**): After PG removal, the tensor veli palatini (TVP) tendon passes through a gap between the buccinator muscle (Bu) and the superior pharyngeal constrictor muscle (SPC) (indicated by arrowheads). Panel (**c**): Lateral view of the hamular notch region, which is located between the maxillary tuberosity (MT) and the pterygoid hamulus (H). Also shown are the thin tendons surrounding the pterygoid hamulus. Panel (**d**): High-magnification view of panel (**c**), depicting three tendons that converge near the H (shown by arrowheads). The Bu is located immediately posterior to the MT (shown in panels **b**-**d**). 1 st : maxillary first molar, 2nd : maxillary second molar

**Fig. 4:**
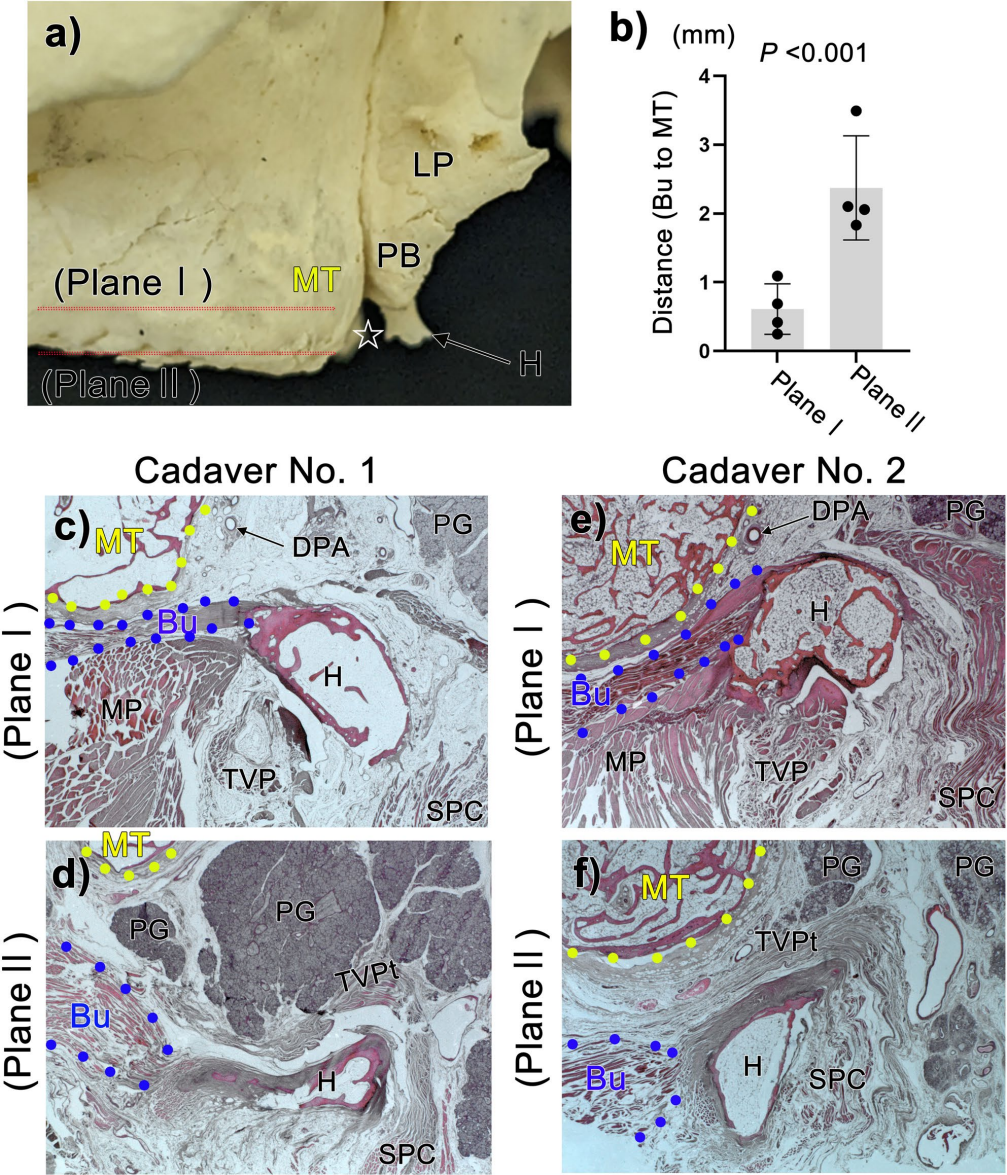
The buccinator muscle in the hamular notch region. Panel (**a**) shows a lateral view photograph of the maxillary tuberosity (MT). Plane I comprises the root of the pterygoid hamulus (H) and the MT, while plane II comprises the tip of the H. Panels (**c**, **d**) show horizontal section photographs of the hamular notch region from cadaver No. 1, while panels (**e**, **f**) show the same region of cadaver No. 2. In plane I, the buccinator muscle (Bu) is located immediately posterior to the MT (panels **c**, **e**). In plane II, the buccinator muscle (Bu) is located posterior to the MT (panels **d**, **f**). Overall, the distance from the Bu to the MT in plane I is shorter than in plane II (plane I, *n* = 4; plane II, *n* = 4) (panel **b**). These differences were evaluated using an unpaired two-tailed Mann-Whitney U test;. Data are expressed as mean ± standard deviation (panel **b**)

**Fig. 5:**
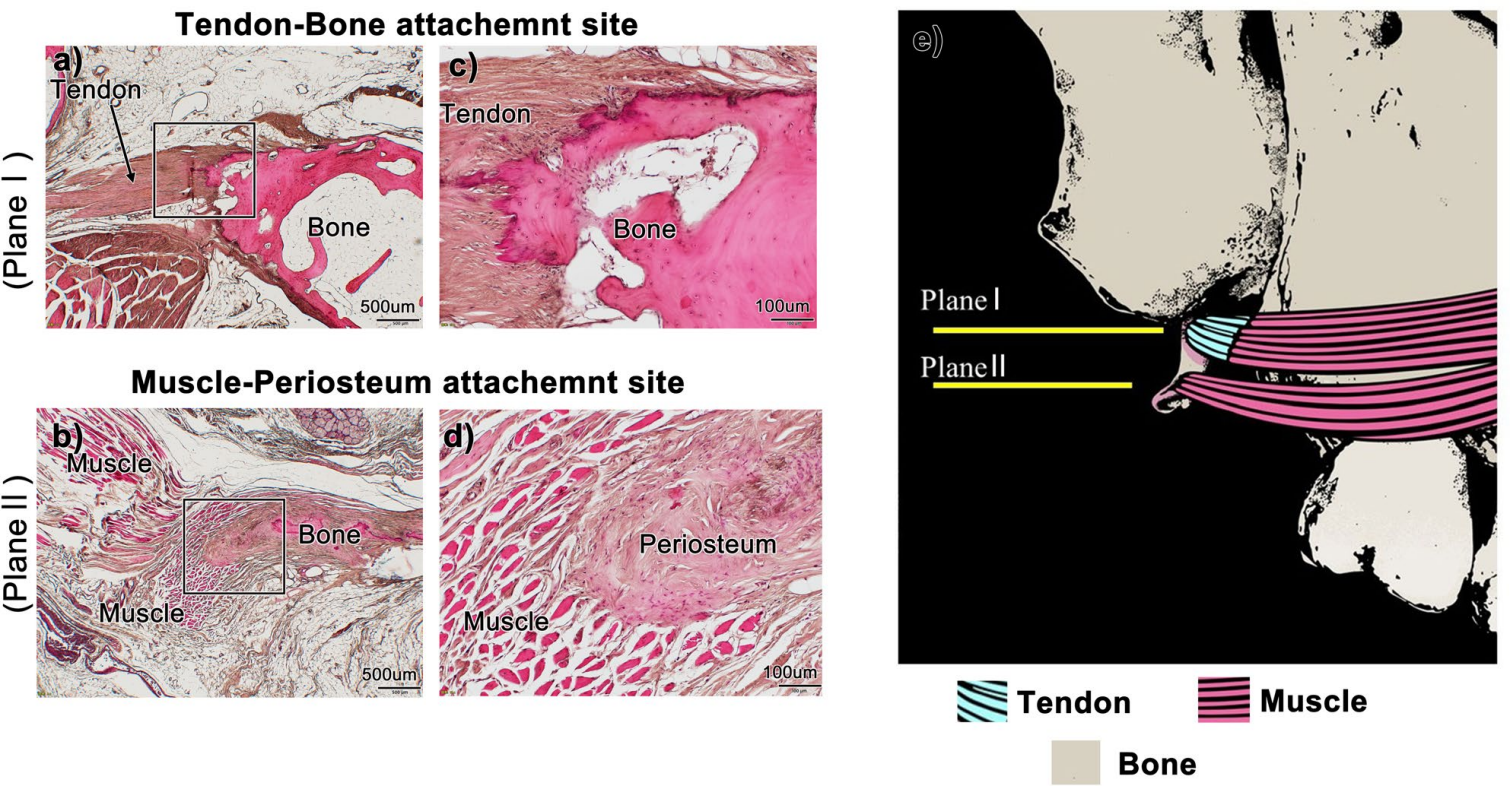
The attachment site of the buccinator muscle within the pterygoid hamulus is divided into two components. Plane I comprises the root of the pterygoid hamulus and the MT, while Plane II comprises the tip of the pterygoid hamulus (see Fig. 4). Panels (**a**-**d**) show the dual origin of the buccinator muscle relative to the pterygoid hamulus. Panels (**c**) and (**d**) show high-magnification views of the squares in panels (**a**) and (**b**). These high-magnification views reveal that at the root of the pterygoid hamulus, the Bu tendon is directly attached to the bone (panel **c**). However, at the tip the buccinator muscle attaches via a thick periosteum without an intervening tendon (panel **d**). Schematic illustration of the hamular notch region (panel **e**). The buccinator muscle is located immediately posterior to the maxillary tuberosity. Therefore, it is likely the first muscle to be damaged in the event of implant perforation. Finally, the buccinator muscle attaches to both the anterior part of the root and the tip of the pterygoid hamulus. Tendon: tendon of the buccinator muscle, Bone: pterygoid hamulus

To ensure anatomical consistency in establishing the sectioning planes for gross and histological analysis, clear definitions of the pterygoid hamulus boundaries were required. Previous morphometric studies report that the hamulus measures approximately 6.4–6.5 mm in total length [19]. Specimens falling within this normative range—thus without evidence of hamular hyperplasia—were selected. Based on this reference length, we defined the sphenoid-anchored proximal portion as the root of the pterygoid hamulus and the freely projecting distal end as the tip. These standardized definitions provided a consistent basis for orienting both gross anatomical sectioning and subsequent histological evaluation.

For micro-computed tomography (micro-CT) images, the hamular notch region and its surrounding tissues were removed from 31 cadavers (15 males and 16 females, aged 70–92 years at death). The number of remaining teeth in the maxillary molar regions was recorded, with third molars excluded (Tables 1 and 2). Samples were then scanned using a micro-CT system (HMX-225 Actis4; Tesco Co., Tokyo, Japan) under the following imaging conditions: tube voltage: 100 kV; tube current: 70 µA; magnification factor: 2.5×; slice width: 5 μm; matrix size: 512 × 512; slice pitch: 50 μm; and voxel size of 140 × 140 × 50 μm. The imaging intensifier was 4 inches in size and contained a 1-inch 16-bit CCD camera with 1024 × 1024 scanning lines. This generated 1200 raw images. Two-dimensional slice data were then prepared using the back-projection method. Images were then constructed using volume rendering of slice data as implemented using three-dimensional (3D) imaging software (VG Studio, Volume Graphics, Heidelberg, Germany) to observe the bone structure present in each image.

**Table 1:**
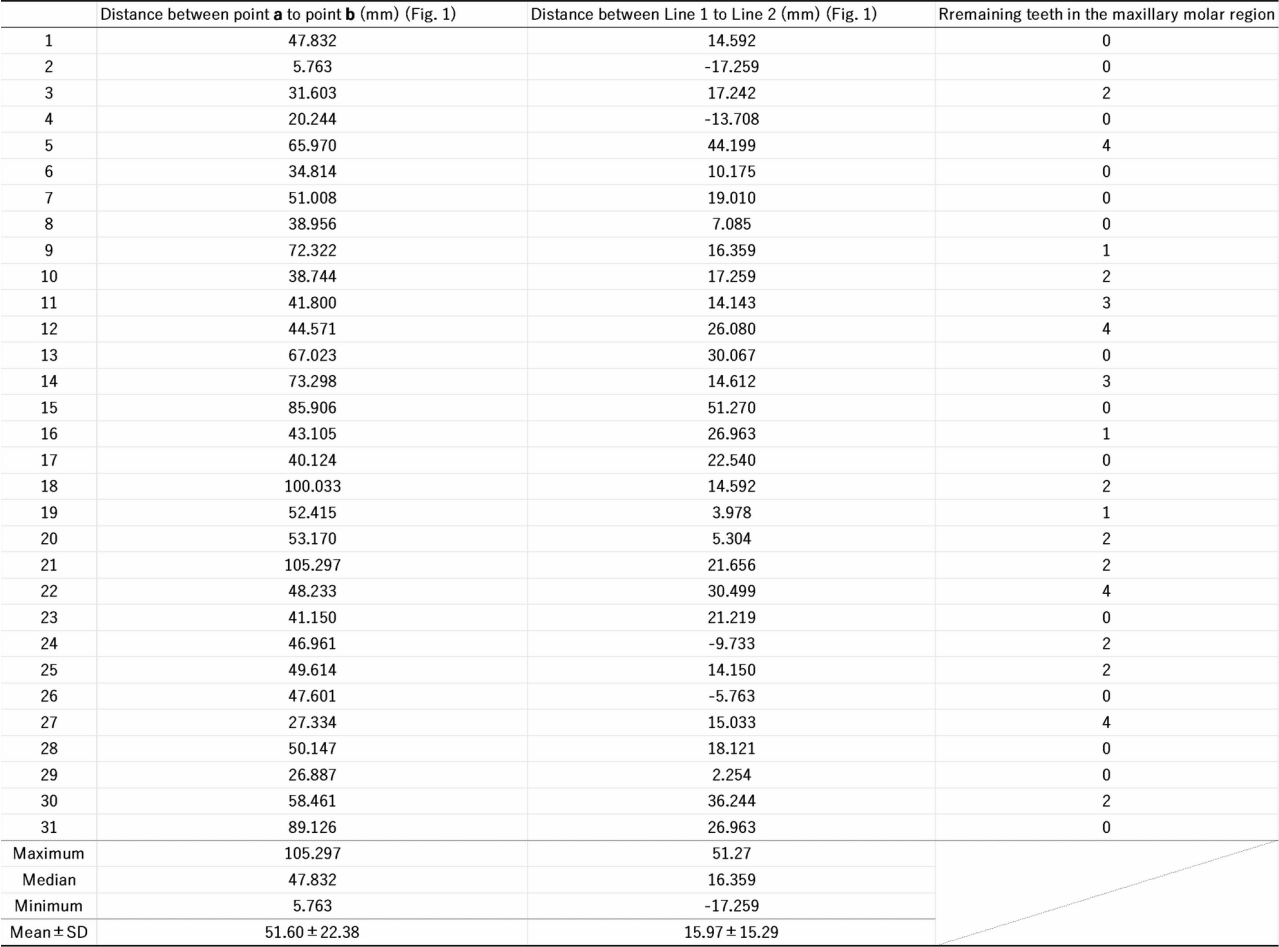
Distance measurement between the maxillary tuberosity surrounding structures

**Table 2:**
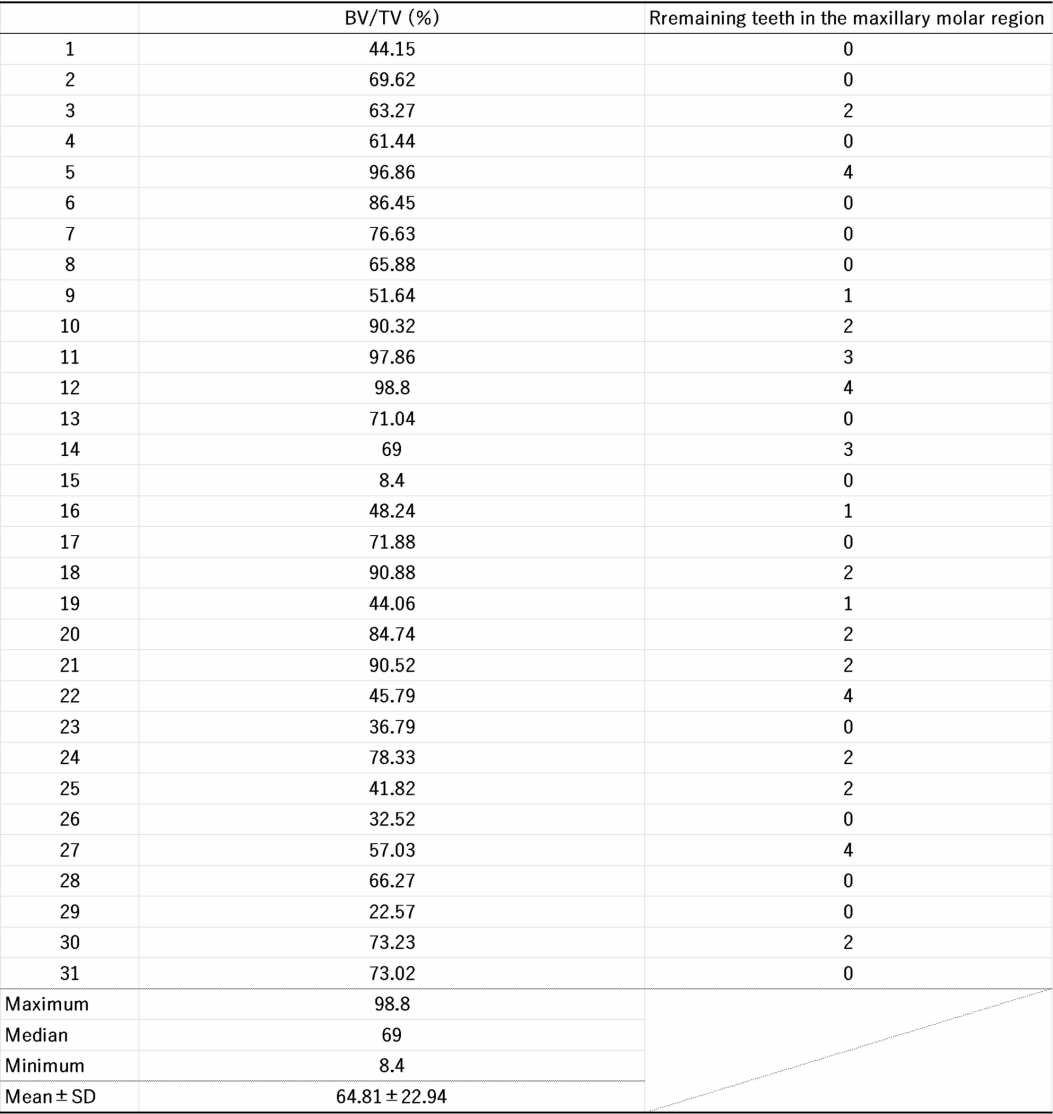
Bone volume (BV/TV) in the maxillary tuberosity

To accurately determine the height of the maxillary tuberosity, we measured: (1) the shortest distance between the posterior point of the maxillary tuberosity and the inferior margin of the maxillary sinus (Fig. 2a, b); and (2) the difference in height between the maxillary tuberosity and the hamulus (Fig. 2c, d). All images thus obtained were analyzed using Image-Pro 148 (Media Cybernetics, MD, United States). Next, bone morphometry was analyzed by using a 3D morphometry system (TRI/3D-BON, Ratoc, Tokyo, Japan) to determine the bone volume fraction (BV/TV; %).

All statistical analyses were conducted using EZR (Saitama Medical Center, Jichi Medical University, Saitama, Japan), a GUI for R (The R Foundation for Statistical Computing, Vienna, Austria). For all analyses, the threshold of statistical significance was set to *P* < 0.05.

## Results

### The maxillary tuberosity is not the origin of most muscles

We first observed that the Bu and medial pterygoid muscles were located posterior to the alveolar part of the maxillary tuberosity (Fig. 1b–e). On the level of the maxillary sinus, the pterygoid process of the sphenoid bone served as the origin for the medial and lateral pterygoid muscles, while only the Bu was weakly attached to the lateral wall of the maxillary sinus in the maxillary tuberosity region (Fig. 1f). Therefore, our analyses showed that most muscles did not attach directly to the maxillary tuberosity. This suggests that muscle function may not significantly influence the maintenance of bone homeostasis in this region.

### The morphology of the maxillary tuberosity varies considerably

If the maxillary tuberosity does not influence muscle contraction force, its morphology may exhibit considerable individual variability due to factors such as tooth loss and osteoporosis. To validate this hypothesis we first measured: (1) the shortest distance between the posterior point of the maxillary tuberosity and the inferior margin of the maxillary sinus (Fig. 2a, b), and (2) the height difference between the maxillary tuberosity and the hamulus (Fig. 2c, d). The former parameter averaged 51.60 ± 22.38 mm, and ranged from 5.76 mm to 105.30 mm with a median of 47.83 mm. The difference in height averaged 15.97 ± 15.29 mm, ranging from − 17.26 mm to 51.27 mm with a median of 16.36 mm (Fig. 2e; Table 1). Moreover, the average BV/TV in the maxillary tuberosity was 64.8 ± 22.9% (Table 2), and ranged from 8.4% to 98.9% with a median of 69.0% (Fig. 2f, g, h; Table 2). Moreover, it has been reported that a low BV/TV in the maxillary tuberosity, coupled with a large height difference between the maxillary tuberosity and the hamulus (as shown in Fig. 2c, g), may increase the risk of implant perforation along its posterior edge [20].

### The buccinator muscle is located posterior to the maxillary tuberosity

Next, we used gross anatomical analysis to identify the topographical relationships between the maxillary tuberosity and the Bu. First, palatine glands were observed on the hard and soft palates (Fig. 3a). After removal of the palatine glands, the tendon of the tensor veli palatini muscle was visible deep between the maxillary tuberosity and the hamulus (Fig. 3b). Importantly, the tensor veli palatini tendon passes through a gap between the Bu and the superior pharyngeal constrictor (Fig. 3b, arrow heads). The Bu bundle was situated posterior to the maxillary tuberosity (Fig. 3c) and appeared to attach to the hamulus (Fig. 3d). Finally, the tendons of the tensor veli palatini, Bu, and superior pharyngeal constrictor were found to converge near the pterygoid hamulus (Fig. 3d) and appeared to attach to it.

### The buccinator muscle May be damaged if pterygoid implants perforate the maxillary tuberosity

Since the Bu is located posterior to the maxillary tuberosity, perforation of this region by pterygoid implants is likely to cause damage. We investigated this risk via histological analysis of the posterior region of the maxillary tuberosity in which we measured the distance from the maxillary tuberosity to the Bu. In brief, sections were prepared in two planes. Plane I included the root of the pterygoid hamulus, while Plane II included the tip of the pterygoid hamulus (Fig. 4a). We presented data from cadavers No.1 and No.2, which were among the four cadavers histologically studied (Fig. 4c-f). In Plane I, the Bu was located immediately posterior to the maxillary tuberosity (0.61 ± 0.367 mm) (Fig. 4b, c, e). In Plane II, the presence of palatine glands or loose connective tis sue immediately posterior to the maxillary tuberosity caused the Bu to be further from the maxillary tuberosity (2.37 ± 0.76 mm) (Fig. 4b, d, f). Moreover, we observed a significant difference was observed in the distance between Plane I and Plane II (*p* < 0.001) (Fig. 2b). These findings indicate that, in Plane I, the buccinator muscle lies immediately posterior to the maxillary tuberosity and is positioned within a minimal soft-tissue buffer zone (approximately 0.6 mm). Accordingly, perforation of the posterior wall in this plane would place the muscle in direct proximity to the implant trajectory.

### The attachment site of the buccinator muscle within the pterygoid Hamulus is divided into two components

In histological sections from all four cadavers (No.1–4), the Bu was found to attach to the pterygoid hamulus. We examined two distinct attachment sites, i.e., at the root and the tip of the pterygoid hamulus (Fig. 5a–d), to determine how the Bu attaches to the hamulus. Higher-magnification views revealed that at the root of the pterygoid hamulus the buccinator tendon directly attaches to the bone (Plane I) (Fig. 5a, c). In contrast, at the tip, the Bu attaches via a thick periosteum without an intervening tendon (Plane II) (Fig. 5b, d). Consequently, damage to the Bu tendon adjacent to the root of the pterygoid hamulus may result in significant functional impairment due to the loss of stable force anchorage.

## Discussion

In this study, we demonstrated that the Bu attaches to the pterygoid hamulus at two distinct sites, i.e., in the root and at the tip (Fig. 5e). Notably, the muscle bundle of the Bu that attaches to the root of the pterygoid hamulus is also located very close to the maxillary tuberosity (Plane I; 0.61 ± 0.367 mm). Therefore, we conclude that dental surgeons must exercise extreme caution when placing pterygoid implants, and should aim to avoid perforating the posterior wall of the maxillary tuberosity.

Most researchers have divided entheses (tendon-bone interface) into fibrous and fibrocartilaginous types based on whether fibrocartilage cells [21–24]. We identified the dual attachment sites of the Bu in the pterygoid hamulus. Since no fibrocartilage cells appeared at these attachment sites, we defined them as fibrous enthesis. In general, the dual attachment sites may reflect functional specialization: the tendinous attachment at the root likely provides a strong and rigid anchor for efficient force transmission during oral activities such as mastication and speech [23]. In contrast, the periosteal attachment at the tip may facilitate a more compliant, flexible interface that enables fine-tuned movement or stabilization under variable loading conditions [23]. At present, relatively few instances of a dual-modal attachment structure for a single muscle attaching to a single bone are known. Moreover, surgical disruption of the tendinous portion may lead to functional impairment due to the loss of stable force anchorage.

Overall, a variety of oral and facial impairments may result from dysfunction of the buccinator muscle. For example, the Bu plays a critical role in supporting the cheek during mastication and maintaining the alignment of the food bolus within the dental arch [25], and certain dysfunctions may lead to food accumulation in the oral vestibule, inefficient chewing, and/or mucosal injury from inadvertent biting of the cheek. Electromyographic studies have shown that the buccinator, together with the orbicularis oris, is involved during articulation of vowels such as “o” and “u,” underscoring its importance for phonation [26]. In addition, Shiratori et al. [27] demonstrated that the buccinator contributes to lip closure by pulling the corners of the mouth, which is critical for speech clarity, producing facial expressions, and general oral continence. Impairment of this mechanism may result in slurred speech, drooling, and/or difficulty with tasks such as whistling or using a straw.

In 1985, Lekholm and Zarb classified bone quality into four categories based on radiographic assessment and the resistance sensed by surgeons during implant site preparation [28]. BV/TV is the parameter that best reflects changes in bone mass on the microstructural level [29]. It can therefore serve as an objective indicator of BMD within the implant region, thereby playing a critical role in determining the initial stability of an implant [30]. Given these advantages, we investigated BV/TV in the maxillary tuberosity. We also noted that systemic factors such as aging, osteoporosis, and hormonal imbalances can contribute to a reduction in BV/TV [31, 32]. Moreover, in the jawbone local factors such as tooth loss, long-term denture use, and chronic periodontal disease further exacerbate such declines [33, 34]. In the present study, we observed considerable individual variability in the BV/TV values of the maxillary tuberosity. When BV/TV is low in the maxillary tuberosity, the resistance during implant insertion is reduced, resulting in an increased risk of perforating the posterior wall. Moreover, the bone density of the pterygoid area is generally greater than in the maxillary tuberosity [9]. Therefore, even in patients with poor maxillary tuberosity bone quality, implants extending into the pterygoid process can achieve reliable primary stability [9].

Previous anatomical works have proposed that the buccinator extends posteriorly toward the pterygomaxillary region, yet none have provided a morphological explanation for the discrepancies among classical descriptions. Nishi (1974) reported a broad extension of buccinator fibers toward the pterygoid process [2], whereas *Gray’s Anatomy* described only a thin tendinous band bridging the maxilla and hamulus [3]. Our identification of two structurally distinct attachment modes— a tendinous insertion at the hamular root and a periosteal connection at the tip—offers a unifying interpretation of these conflicting accounts. The slender tendinous band described in classical texts corresponds well to the root attachment, while the broader posterior extension reported by Nishi can be attributed to the periosteal tip connection revealed in the present study.

More recent literature, such as the report by Iwanaga et al. (2022) on a “hamular part” of the buccinator, further implies a relationship between the muscle and the hamulus [4]. However, that study did not differentiate between attachment types nor include histological evaluation, leaving the structural basis of this connection unresolved. The present findings refine this concept by demonstrating that the buccinator engages the hamulus through two anatomically and histologically distinct interfaces.

Functional and imaging studies have emphasized the buccinator’s involvement in shaping the posterior vestibule and modulating forces in the maxillary tuberosity–hamulus region [35, 36]. Animal experiments by Dutra et al. [37] also support a mechanical role for the buccinator in the posterior alveolar process. While these studies collectively highlight the buccinator’s functional significance, they do not address its precise insertional anatomy. The dual-attachment pattern revealed here provides the structural foundation necessary to interpret these functional observations.

Despite the clinical importance of the hamular region in pterygoid implant placement, authoritative implantology textbooks provide limited discussion of soft-tissue attachments. *Clinical Anatomy for Oral Implantology* [38] describes the hamulus primarily as a pulley for the tensor veli palatini and notes adjacent fibers without assigning them to the buccinator. Similarly, *Contemporary Implant Dentistry* [39] and *Implants and Oral Rehabilitation of the Atrophic Maxilla* [40] detail pterygomaxillary anatomy for implant planning but do not describe buccinator–hamulus attachments. The absence of such descriptions in surgical literature underscores the novelty and clinical relevance of the two distinct buccinator insertions identified in this study.

This study has several methodological limitations. First, the sample size was relatively small and consisted primarily of elderly cadavers. This reflects the inherent difficulty of obtaining large numbers of cadaveric specimens and preparing extensive tissue blocks for histological and micro-CT analysis. Consequently, the age distribution of the sample may limit the generalizability of the findings to younger populations. Nonetheless, this age group represents the predominant demographic receiving pterygoid implants, largely due to the high prevalence of posterior maxillary atrophy and long-term denture-related remodeling in older adults. Second, factors such as sex differences, bilateral asymmetry, bone quality, and dental or prosthetic history were not systematically assessed. Although considerable anatomical variability exists in the maxillary tuberosity–hamular region, prior studies have not identified consistent sex-specific differences, and our histological observations suggest that the fundamental architecture of soft-tissue attachments around the hamulus may be relatively stable across individuals. However, unrecognized variation related to sex, bone density, or dental history cannot be completely excluded. Third, cadaver selection was limited to individuals who died of ischemic cardiac or cerebral diseases and had no gross pathological abnormalities. Although these causes of death are not known to affect masticatory muscle or perioral soft-tissue attachment anatomy, it remains possible that systemic or age-related factors, undetectable at the macroscopic level, could introduce bias. Future studies involving larger and more diverse samples, bilateral evaluations, and stratification by bone quality, dental status, and prosthetic history will be essential to further validate and extend the anatomical insights presented in this study.

## Conclusion

A detailed understanding of the posterior maxillary region—including muscular attachments and maxillary tuberosity morphology—is essential for safe and predictable pterygoid implant placement. The present study suggests that recognition of the buccinator’s dual attachment to the pterygoid hamulus, together with awareness of anatomical variability in this region, may contribute to improved surgical planning. However, because no clinical or surgical outcome data were analyzed, these implications remain theoretical. Future clinical, biomechanical, and imaging-based studies will be needed to determine whether these anatomical relationships influence optimal implant trajectory, minimize soft-tissue injury, and improve long-term functional outcomes.

## Data Availability

All data sets used and analyzed during the current study are available from the corresponding author upon reasonable request.

## Abbreviations

Apo: Palatine aponeurosis
Bu: Buccinator muscle
But: Buccinator tendon
DPA: Descending palatine artery
GPC: Greater palatine canal
H: Pterygoid hamulus
LP: Lateral pterygoid muscle
LPC: Lesser palatine canal
MP: Medial pterygoid muscle
MT: Maxillary tuberosity
MX: Palatine bone
PG: Palatine gland
SPC: Superior pharyngeal constrictor muscle
TVP: Tensor veli palatini muscle
BV/TV: Bone volume/total volume
BMD: Bone mineral density
CBCT: Cone beam computed tomography
